# Multiomics characterization of fatty acid metabolism for the clinical management of hepatocellular carcinoma

**DOI:** 10.1038/s41598-023-50156-7

**Published:** 2023-12-18

**Authors:** Xin Huang, Benzhe Su, Mengjun Li, Yang Zhou, Xinyu He

**Affiliations:** 1 School of Artificial Intelligence, Anshan Normal University, Pingan Street, Anshan, 114007 Liaoning China; 2 Biomedical Engineering Postdoctoral Research Station, Dalian University of Technology, Dalian, Liaoning China; 3 Postdoctoral Workstation of Dalian Yongjia Electronic Technology Co., Ltd, Dalian, Liaoning China; 4 School of Computer Science and Technology, Dalian University of Technology, Dalian, Liaoning China; 5 Ningbo Institute of Innovation for Combined Medicine and Engineering, Ningbo Medical Center Li Huili Hospital, Ningbo, Zhejiang China; 6 School of Computer and Information Technology, Liaoning Normal University, Dalian, Liaoning China

**Keywords:** Biological techniques, Cancer, Biomarkers

## Abstract

Hepatocellular carcinoma (HCC) is a prevalent malignancy and there is a lack of effective biomarkers for HCC diagnosis. Living organisms are complex, and different omics molecules interact with each other to implement various biological functions. Genomics and metabolomics, which are the top and bottom of systems biology, play an important role in HCC clinical management. Fatty acid metabolism is associated with malignancy, prognosis, and immune phenotype in cancer, which is a potential hallmark in malignant tumors. In this study, the genes and metabolites related to fatty acid metabolism were thoroughly investigated by a dynamic network construction algorithm named EWS-DDA for the early diagnosis and prognosis of HCC. Three gene ratios and eight metabolite ratios were identified by EWS-DDA as potential biomarkers for HCC clinical management. Further analysis using biological analysis, statistical analysis and document validation in the discovery and validation sets suggested that the selected potential biomarkers had great clinical prognostic value and helped to achieve effective early diagnosis of HCC. Experimental results suggested that in-depth evaluation of fatty acid metabolism from different omics viewpoints can facilitate the further understanding of pathological alterations associated with HCC characteristics, improving the performance of early diagnosis and clinical prognosis.

## Introduction

Hepatocellular carcinoma (HCC) is the third leading cause of cancer-related deaths, which is a serious barrier to increasing life expectancy and brings enormous economic burdens for both families and society as a whole^1,2^. Late presentation at advanced stages, early metastasis and tumor recurrence after surgical resection always lead to a high mortality rate^3–5^. In fact, when patients are diagnosed with HCC in the early stage, intervention can be effectively performed to improve clinical management performance, especially the survival rate^6,7^. However, HCC development involves the interactions of various extrinsic and intrinsic factors, including genetic, metabolic and environmental factors, which bring enormous challenges for early HCC diagnosis and precise prognosis. To date, the pathogenic mechanisms of HCC are not quite clear, so there is a lack of effective biomarkers for HCC clinical management^8^.

Genomics and metabolomics, which are the top and bottom of systems biology, play an important role in cancer clinical management, including early diagnosis and precise prognosis^9,10^. Cells become cancer cells largely because of abnormal changes in genes. This progression is considerably complex and involves many mutations in biological activities related to cell growth and division. The dysfunction of genes can result in the dysfunction of RNA and proteins, ultimately resulting in the dysfunction of metabolites. Metabolomics, the study of the final downstream products of gene expression, is widely applied in disease diagnosis and personalized medicine in the post-genomics era. In metabolomics, disease development can be reflected by dynamic changes in metabolite concentrations, thereby contributing to clinical medicine studies. The relationships between metabolomics and genomics are extremely close in living organisms. Therefore, a previous study suggested that the integration of the data from metabolomics and genomics for cancer studies is a better way to deeply understand the complex biochemical processes of cancer development^11^.

Because living organisms experience metabolic disorders with cancerization onset, cancer is viewed as a metabolic disease. For example, the metabolism of fatty acids in malignant tumor cells is significantly different from that in normal cells^12^. Fatty acid metabolism plays an important role in various biological activities, including cell membrane formation, energy storage, and signaling molecule generation in oncogenesis^13,14^. However, a living organism is a complex biological network in which different omics molecules interact with each other for the implementation of various physiological functions^15^. For example, human cancer molecules can affect the metabolic process, in which some cancer-related genes and metabolites participate together in fatty acid metabolism^16,17^. Therefore, in-depth exploration of changes in fatty acid metabolism-related genes and metabolites can provide novel insights for a better understanding of the pathogenetic mechanism of HCC. Some studies have investigated a single regulator of fatty acid metabolism in HCC from the viewpoints of genomics or metabolomics; however, to our knowledge, the integrated roles of genes and metabolites related to fatty acid metabolism are unknown.

Research has shown that the initiation and progression of HCC is a dynamic process involving oncogenes and tumor suppressors forming pathways and networks^18^. In addition, relationships among different omics molecules undergo stepwise changes with the gradual deterioration of liver function, and some change trends only occur at the HCC early stage rather than other stages of HCC development. Hence, network warning signals representing the occurrence of early-stage HCC are likely to exist and could be used for the early detection of HCC in high-risk populations. This study adopted our bioinformatics network analysis method, namely, the early warning signals based on a data-driven approach (EWS-DDA)^19^, to identify early diagnosis biomarkers by the exploration of dynamic network information of multiomics HCC data (genes and metabolites) from both clinical samples and animal models. In systems biology, ratio relationships between molecules can represent the result of an assumed pathway reaction in which a molecule is converted into another via single or multiple reaction paths^20^. Therefore, EWS-DDA measures dynamic expression changes in molecular ratios during cancer development for network construction and uses a data-driven approach for the identification of early network warning signals. In EWS-DDA, the shrunken centroid was employed to infer the *t* statistic to measure dynamic changes in molecular ratios during the processes of cancer initiation and progression. Compared with other traditional network representation learning methods, two major advantages of EWS-DDA are the study of mechanisms in a biochemical manner by reviewing the pathway reactions within the network and the definition of early diagnosis biomarkers by means of a data-driven approach^19^.

In this study, we provided novel insights into the molecular pathology of HCC development by means of dynamics and network analysis, in which genes and metabolites related to fatty acid metabolism were simultaneously investigated for the early diagnosis and precise prognosis of HCC. The biological roles of crucial genes and metabolites related to fatty acid metabolic processes were revealed during the initiation and progression processes of HCC at a network level, which can facilitate a better understanding of HCC pathogenic mechanisms. Figure S1 exhibits the comprehensive details on the procedures of this study.

## Results

### Changes in the topological structure of the gene networks in HCC development

**Figure 1:**
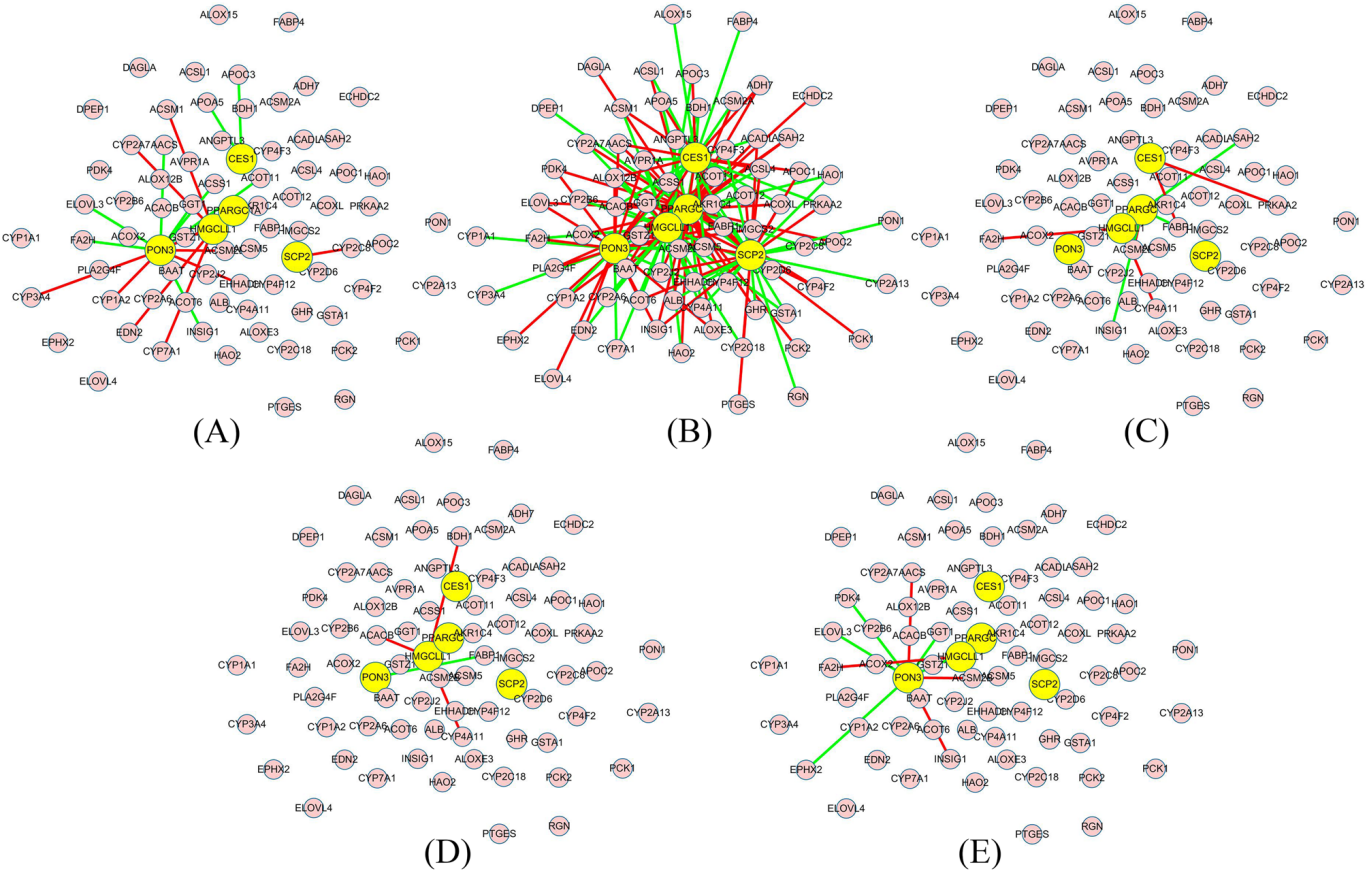
Changes in the topological structure of *SG*_I_ during the process of HCC progression. (**A**–**E**) Topological structures of *SG*_I_ in the normal, stage I, stage II, stage III and stage IV groups.

Due to five different stage samples contained in the discovery set of genomics datasets, EWS-DDA constructed five dynamic networks, namely, *G*_N_, *G*_I_, *G*_II_, *G*_III_, and *G*_IV_, to represent changes in biological activities associated with fatty acid metabolism in the process of HCC initiation and progression (see Figure S2). In network *G*_*k*_ (*k* = N, I, II, III and IV) constructed by EWS-DDA, if *d*_*ijk*_ is larger than or equal to 0.6 at the *k*th stage, *f*_*i*_ and *f*_*j*_ are linked by a red edge; if *d*_*ijk*_ is less than or equal to − 0.6 at the *k*th stage, *f*_*i*_ and *f*_*j*_ are linked by a green edge. Stage I HCC is the early stage, which is the best period to prevent further deterioration. Therefore, *G*_I_ was explored in depth, and nodes in *G*_I_ were ranked in descending order according to their degrees. The top five nodes and their first linking nodes were extracted to construct the crucial subnetwork *SG*_I_, which can function as a representative of pathway reactions in the early stage of HCC. Figure 1 shows the changes in the topological structure of *SG*_I_ in HCC development. The relationships of genes in *SG*_I_ are markedly different between stage I and the other stages. For example, compared with those in the other stages in the process of HCC development, genes in *SG*_I_ were closely related to each other and had definite relationships in stage I. The change trends in topological structures of *SG*_I_ suggested that it has great power for the indication of HCC onset at the network level. Therefore, *SG*_I_ can be viewed as a network warning signal that represents the deterioration of HCC and aids in early clinical diagnosis.

### Potential biomarker discovery from the HCC genomics datasets

**Figure 2:**
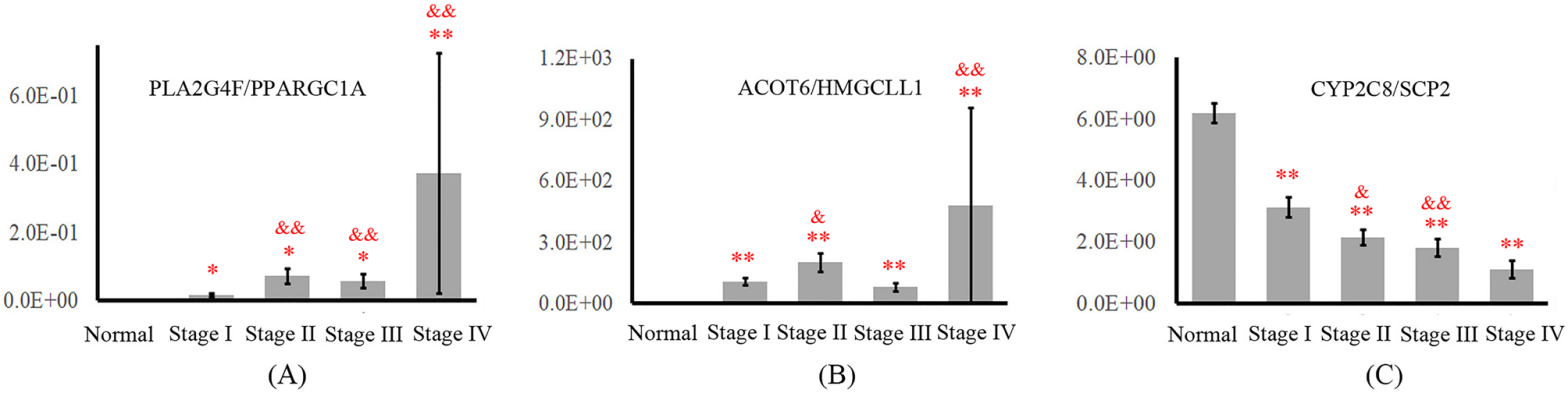
Changes in the expression of PLA2G4F/PPARGC1A, ACOT6/HMGCLL1 and CYP2C8/SCP2 during HCC development. (**A**–**C**) The gene expression trajectories (mean ± S.E.) of PLA2G4F/PPARGC1A, ACOT6/HMGCLL1 and CYP2C8/SCP2. The * indicates statistical significance between the corresponding group and the normal group. The & indicates statistical significance between the corresponding group and stage I group.

PPARGC1A, SCP2, PON3, CES1 and HMGCLL1 were the top five nodes with the largest degree in *SG*_I_, which had a strong ability to reflect physiological and pathological changes in living organisms in the early stage of HCC. Because EWS-DDA explored the ratio relationship between molecules for dynamic network construction, ratios inferred by the top five genes and their first linking nodes were studied in depth. The statistical analysis *t* test was used to further validate the discriminative ability of potential biomarkers. Due to the importance of stage I HCC in early diagnosis, gene ratios that had at least 2 significantly different results (i.e., *p *value < 0.05) between the stage I group and the other three HCC groups (i.e., stage II, stage III and stage IV) were viewed as potential biomarkers. In addition, the selected potential biomarkers should have significant differences between the normal group and each of the four HCC groups, thereby contributing to HCC diagnosis. Six gene ratios simultaneously met all of the above statistical analysis conditions (see Table S1). Finally, according to the change trends in gene expression trajectories during HCC development, PLA2G4F/PPARGC1A, ACOT6/HMGCLL1 and CYP2C8/SCP2 were defined as crucial information for early diagnosis (see Fig. 2). PLA2G4F/PPARGC1A and ACOT6/HMGCLL1 increased stepwise with the gradual deterioration of liver function, aiding in reflecting the differential changes in biological activities at different HCC stages (see Fig. 2A,B). The level of CYP2C8/SCP2 was decreased stepwise during the initiation and progression of HCC, which also facilitated a better understanding of the pathological alterations associated with HCC characteristics (see Fig. 2C). The results of statistical analysis and expression change trajectory suggested that the three ratios can regularly represent dynamic changes in the underlying system during the process of HCC progression and provide important information for clinical HCC diagnosis.

**Figure 3:**
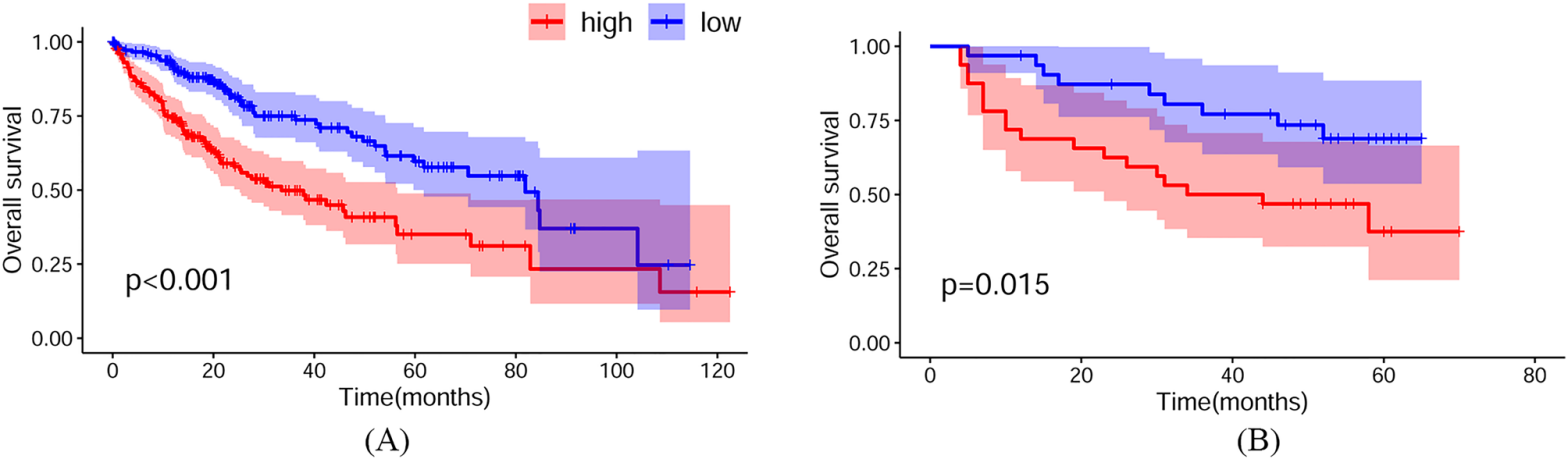
Prognostic performance of the selected gene biomarkers. (**A**) and (**B**) Survival analysis results based on multivariate Cox regression of the 3 selected gene ratios in the discovery set and in validation set 1.

Survival analysis was carried out on the discovery set and validation set 1 to explore the relationship between OS and the hub genes in HCC patients. A log-rank *p* value < 0.05 for comparisons between the low-risk and high-risk groups was regarded as statistically significant. Figure 3A and B show the Kaplan–Meier curve of multivariate Cox regression in the discovery set and validation set 1. HCC patients with high-risk scores in the discovery set had a significantly worse OS than HCC patients with low-risk scores (see Fig. 3A). This result suggested that the selected gene biomarkers were involved in HCC induction and had great power for the assessment of high-risk occupational populations. The significantly different expression in the low- and high-risk score groups was also represented in the HCC patients in validation set 1 (see Fig. 3B). The experimental result from validation set 1 was consistent with that from the discovery set, thereby contributing to the further validation of the prognostic values of these potential biomarkers. The survival analysis of the discovery set and validation set 1 strongly suggested that the selected potential gene biomarkers were closely related to HCC determination and may result in the induction and promotion of HCC.

Then, a binary logistic regression model was constructed based on the selected biomarker panel (PLA2G4F/PPARGC1A, ACOT6/HMGCLL1 and CYP2C8/SCP2) for the measurement of their importance for HCC clinical management. The AUC, specificity, sensitivity, S.E. and 95%CI were determined using the discovery set and validation set 2 to comprehensively investigate the diagnostic ability. In the discovery set (see Table 1), the AUC value of the biomarker panel was 0.949 for distinguishing the model group from the normal group. Due to the imbalance between the normal samples and the model samples in the discovery set, specificity and sensitivity can better reflect the effectiveness of the biomarker panel in clinical application. The ROC model showed that 87.9% of the model samples and 94.0% of the normal samples were correctly distinguished at the best cutoff value in this study. In the clinic, stage I HCC and stage II HCC are the crucial periods for early diagnosis and should be explored in depth. The AUC value of the biomarker panel was 0.937 for distinguishing the normal group from the early-stage group (combining stage I and stage II). In addition, 81.7% of early-stage HCC samples and 98.0% of the normal samples were correctly distinguished at the best cutoff value in the discovery set. The ranges of the 95%CI from the above two experiments were [0.928–0.970] and [0.911–0.964], respectively, which showed the statistical results with high confidence. In validation set 2, for the normal versus model groups in the GSE62232 dataset, the AUC, specificity, sensitivity and S.E. values of the biomarker panel were 0.964, 1.000, 0.889 and 0.019, respectively (see Table 2). The range of the 95%CI was [0.927–1.000], which further validated its precise ability for HCC diagnosis. For the GSE174570 dataset, the biomarker panel also had good performance for the separation between the normal and model groups (i.e., AUC = 0.892, specificity value = 0.860, sensitivity value = 0.895 and S.E. = 0.034). The range of the 95%CI was [0.826–0.958]. The good performance of the gene biomarker panel in the discovery set and validation set 2 suggested its great power for precise HCC diagnosis.

**Table 1:** Performance of different methods in the genomics discovery set.

Data	Methods	AUC	Spe.	Sen.	S.E.	95%CI
N versus M	EWS-DDA	0.949	0.940	0.879	0.011	[0.928–0.970]
	DMNC	0.939	0.856	0.980	0.012	[0.916–0.962]
	DNB-HC	0.795	0.646	0.940	0.023	[0.750–0.841]
	ATSD-DN	0.925	0.847	0.960	0.014	[0.896–0.953]
	MN-PCC	0.710	0.620	1.000	0.054	[0.604–0.817]
N versus E.S	EWS-DDA	0.937	0.980	0.817	0.013	[0.911–0.964]
	DMNC	0.943	0.852	0.980	0.013	[0.918–0.968]
	DNB-HC	0.782	0.623	0.940	0.027	[0.730–0.834]
	ATSD-DN	0.928	0.844	0.960	0.016	[0.898–0.959]
	MN-PCC	0.700	0.620	1.000	0.056	[0.590–0.810]

*N* Normal group, *M* Model group, *E.S* Early group (combining stage I and stage II).

**Table 2:** Performance of different methods in the genomics validation set 2.

Data	Methods	AUC	Spe.	Sen.	S.E.	95%CI
GSE62232	EWS-DDA	0.964	1.000	0.889	0.019	[0.927–1.000]
	DMNC	0.949	0.852	1.000	0.023	[0.904–0.994]
	DNB-HC	0.865	0.778	1.000	0.038	[0.792–0.939]
	ATSD-DN	0.863	0.790	0.900	0.077	[0.712–1.000]
	MN-PCC	0.848	0.975	0.600	0.063	[0.725–0.971]
GSE174570	EWS-DDA	0.892	0.860	0.895	0.034	[0.826–0.958]
	DMNC	0.887	0.754	1.000	0.036	[0.817–0.958]
	DNB-HC	0.787	0.649	0.895	0.045	[0.699–0.875]
	ATSD-DN	0.779	0.719	0.754	0.043	[0.694–0.864]
	MN-PCC	0.579	0.544	0.614	0.054	[0.474–0.684]

### Changes in the topological structure of the metabolic networks in HCC development

**Figure 4:**
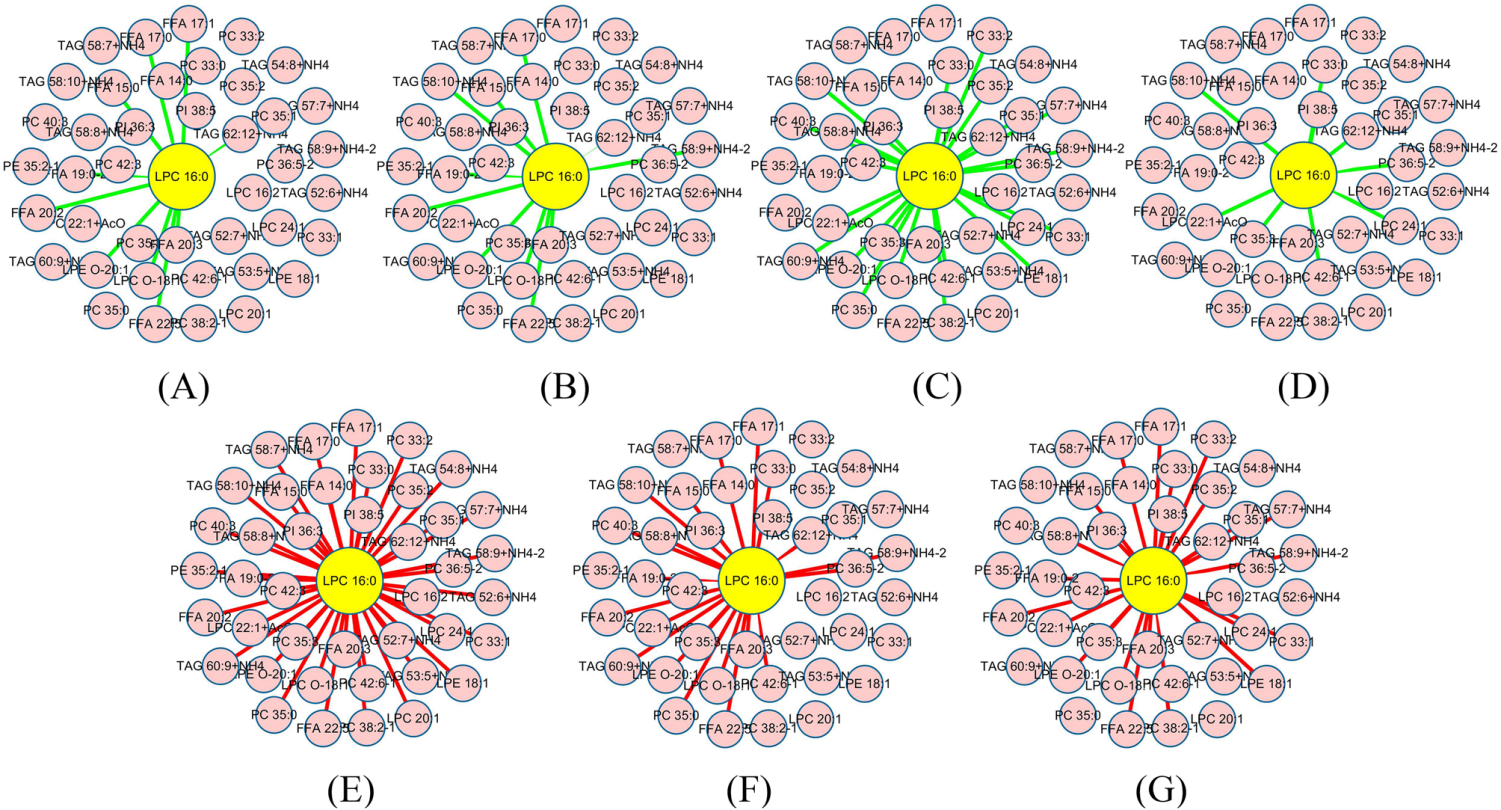
Changes in the topological structure of *SDN-5* during HCC progression. (**A**–**G**) Topological structures of *SDN-5* along the time points.

There were 7 time points in the model group of the metabolomics discovery set. EWS-DDA constructed 7 dynamic networks (*DN*-*i*, 1 ≤ *i* ≤ 7) to characterize the changes in pathway reactions associated with fatty acid metabolism during the initiation and progression of HCC, in which the parameter *ε* (i.e., |*d*_*ijk*_|, *k* = 1, 2,…, 7) was set as 2.3 (see Figure S3). *T*_5_ is the first time point of HCC stage, which is the best period for HCC clinical management; therefore, *DN*-5 was first emphasized for the study of early HCC diagnosis. LPC 16:0 was the node with the largest degree in *DN*-5, which suggests that it can contain the most biological information to diagnose HCC patients at the early stage. The subnetwork *SDN*-5 that was constructed by LPC 16:0 and its first linking nodes was extracted for subsequent network analysis and potential biomarker discovery. The changes in the topological structure of *SDN*-5 during the process of HCC progression are shown in Fig. 4. In EWS-DDA, because ratio relationships between metabolites were considered in the dynamic network construction, the edges in networks can represent the dynamics of circulating metabolites in HCC development. Among the dynamic networks of HCC deterioration, the metabolic network with the most edges can represent the largest difference in the dynamics of circulating metabolites, which has great potential power to imply physiological or pathological abnormalities. Therefore, the stage in which the corresponding network had the most change in topological structure was the crucial period along the time course and the key point for the biological process of liver disease developing from precancer to HCC and continuing to deteriorate. Figure 4 shows that when HCC occurred, the topological structure of *SDN*-5 at time point *T*_5_ significantly differed from that at the other time points. It can be seen that *SDN*-5 had the most edges compared with the other networks during HCC development. In addition, the edges in the other networks at the pre-HCC stages (see Fig. 4A–D) were green. However, when the liver disease developed to the early HCC stage, the edges in the network were red and maintained the state at the whole HCC stage (see Fig. 4E–G). The change trend in topological structure of *SDN*-5 can be applied to indicate liver disease determination and facilitate the understanding of pathological alterations related to fatty acid metabolism. This discovery suggested the validity of the network signal that agreed with HCC development validated by histological examination. The analysis results of the dynamic metabolic network indicated that *SDN*-5 was the crucial prospective network signal representing the onset of HCC, aiding in improving the early precise diagnoses of HCC.

### Potential biomarker discovery from the HCC metabolomics datasets

**Figure 5:**
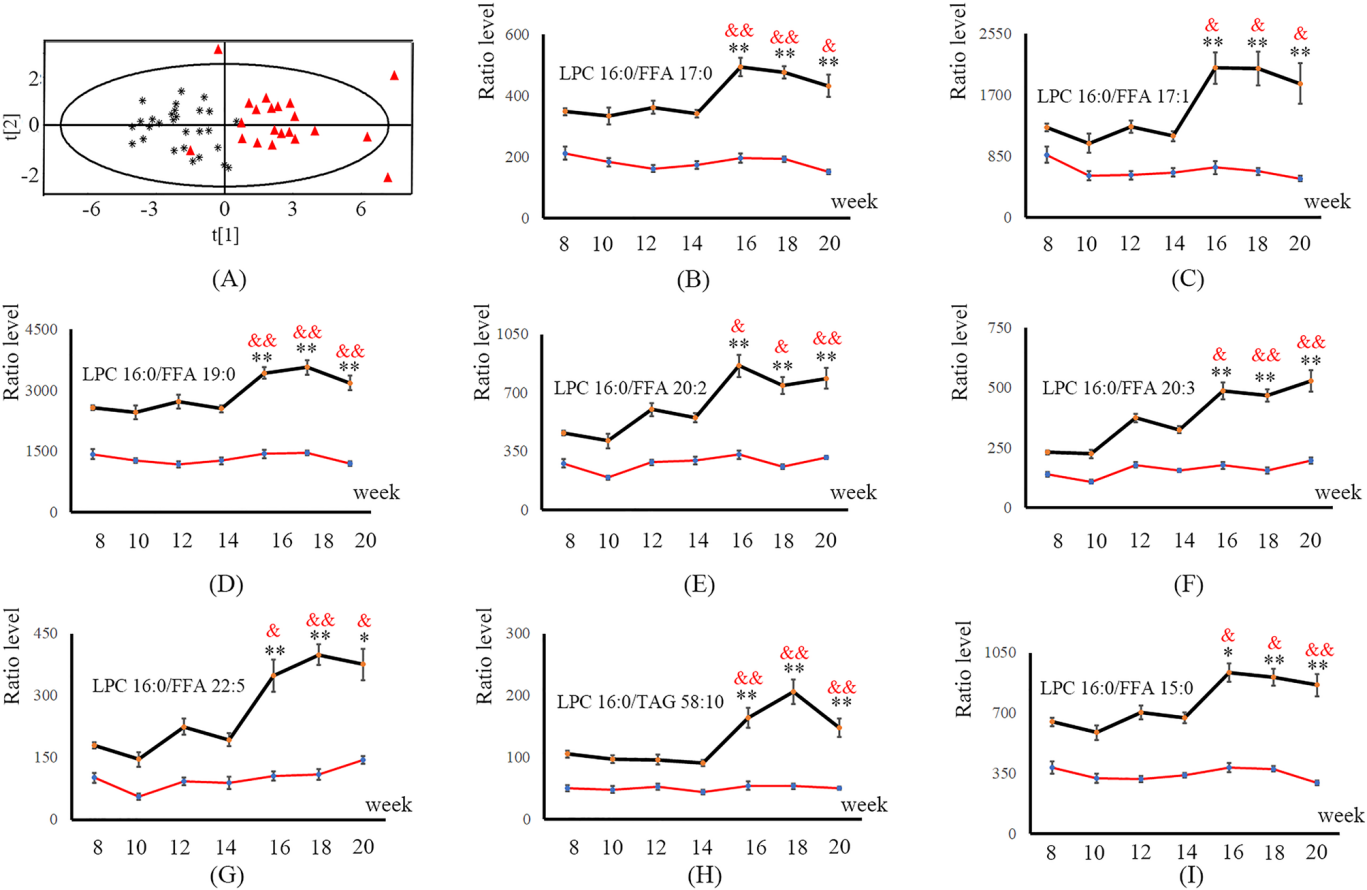
Statistical analysis of the potential metabolite biomarkers. (**A**) PCA score plots based on the results of the selected potential metabolite biomarkers. Black star: pre-HCC samples, red triangle: HCC samples. (**B**–**I**) Metabolic trajectories (the mean ± the S.E.) of LPC 16:0/FFA 17:0, LPC 16:0/FFA 17:1, LPC 16:0/FFA 19:0, LPC 16:0/FFA 20:2, LPC 16:0/FFA 20:3, LPC 16:0/FFA 22:5, LPC 16:0/TAG 58:10 and LPC 16:0/FFA 15:0. Red line: normal group; black line: model group. The red &: significant difference between the typical CIR time point (*T*_4_, week 14) and any time points at the HCC stage (*T*_5_-*T*_7_, weeks 16–20). The black *: significant difference between the control group and model group. * and & indicate *p* < 0.05, ** and && indicate *p* < 0.01.

Metabolite ratios constructed by LPC 16:0 and its first linking nodes in *SDN*-5 were used for subsequent statistical analysis. The univariate evaluation methods, *t* test and paired *t* test, were applied to analyze the significant difference between the model and age-matched control groups at the HCC stage and between *T*_4_ and any time point at the whole HCC stage. Ten metabolite ratios showed significant differences for all univariate evaluations, thereby contributing to the early precise diagnosis of HCC (see Table S2). PCA was performed based on the ten metabolite ratios to validate their ability to separate samples between the HCC and non-HCC groups. The score plot directly shows that the metabolic profiling of all HCC samples had an obvious variation along the first principal compared with non-HCC samples (see Fig. 5A). According to the continuous changes in metabolic trajectories during the process of HCC development, eight metabolite ratios were selected as potential biomarkers for HCC clinical management (see Fig. 5B–I). Compared with the model group, the levels of these eight metabolite ratios in the normal group changed slightly over time. In the model group, the levels of these eight metabolite ratios showed little significant difference at the pre-HCC stage. However, when the liver disease developed to the early HCC stage, the levels of these eight metabolite ratios appeared to increase significantly and maintained a state of high expression throughout the whole HCC stage. Therefore, the change trend in the levels of eight selected metabolite ratios can effectively represent HCC onset. The experimental results suggested that these eight metabolite ratios had great clinical application value for the early precise diagnosis of HCC.

The AUC, specificity, sensitivity, S.E. and 95%CI values of the binary logistic regression model constructed by the eight selected metabolite ratios in the metabolomics discovery and validation sets are shown in Table 3 and Table 4, respectively. For the case of HCC versus non-HCC (combining H samples and CIR samples) in the discovery set, the biomarker panel can distinguish HCC samples from non-HCC samples with an AUC of 0.981. In addition, 90.5% of HCC samples and 100.0% of non-HCC samples were correctly diagnosed at the best cutoff value. The S.E. of the biomarker panel was 0.016, and the range of the 95%CI was [0.950–1.000]. For the case of HCC versus CIR, the AUC, specificity and sensitivity values were 0.977, 1.000 and 0.905, respectively. The S.E. was 0.019, and the range of the 95%CI was [0.940–1.000]. In the validation set, the biomarker panel distinguished HCC samples from non-HCC samples with an AUC of 0.983. The specificity, sensitivity and S.E. values were 0.917, 1.000 and 0.017, respectively. The range of the 95%CI was [0.949–1.000]. For the case of HCC versus CIR, the AUC, specificity, sensitivity, S.E. and the range of the 95%CI values were the best performance.

**Table 3:** Performance of different methods in the metabolomics discovery set.

Data	Methods	AUC	Spe.	Sen.	S.E.	95%CI
HCC versus non-HCC	EWS-DDA	0.981	1.000	0.905	0.016	[0.950–1.000]
	DMNC	0.891	0.810	0.893	0.048	[0.797–0.985]
	DNB-HC	0.912	0.857	0.929	0.047	[0.819–1.000]
	ATSD-DN	0.959	0.905	0.929	0.028	[0.904–1.000]
	MN-PCC	0.820	0.714	0.857	0.063	[0.697–0.942]
HCC versus CIR	EWS-DDA	0.977	1.000	0.905	0.019	[0.940–1.000]
	DMNC	0.893	0.857	0. 857	0.056	[0.783–1.000]
	DNB-HC	0.916	0.857	0.905	0.046	[0.826–1.000]
	ATSD-DN	0.966	0.952	0.905	0.024	[0.919–1.000]
	MN-PCC	0.800	0.714	0.857	0.070	[0.664–0.937]

**Table 4:** Performance of different methods in the metabolomics validation set.

Data	Methods	AUC	Spe.	Sen.	S.E.	95%CI
HCC versus non-HCC	EWS-DDA	0.983	0.917	1.000	0.017	[0.949–1.000]
	DMNC	0.892	0.833	0.833	0.053	[0.788–0.996]
	DNB-HC	0.941	0.917	0.833	0.037	[0.868–1.000]
	ATSD-DN	0.972	0.917	1.000	0.029	[0.916–1.000]
	MN-PCC	0.517	0.333	0.857	0.114	[0.294–0.741]
HCC versus CIR	EWS-DDA	1.000	1.000	1.000	0.000	[1.000–1.000]
	DMNC	0.866	0.833	0.778	0.065	[0.738–0.994]
	DNB-HC	0.935	1.000	0.722	0.042	[0.854–1.000]
	ATSD-DN	0.986	0.917	1.000	0.017	[0.953–1.000]
	MN-PCC	0.523	0.250	0.944	0.115	[0.298–0.748]

The EWS-DDA algorithm was compared with other network algorithms including DMNC, DNB-HC, ATSD-DN and MN-PCC. (see Tables 1, 2, 3 and 4). For the case of N versus M in the genomics discovery set, EWS-DDA had the highest ROC and the best values of 95%CI, thereby validating its better predictive accuracy. Compared with other network algorithms, EWS-DDA had the lowest S.E., which suggested its robustness for HCC diagnosis. The sum of sensitivity and specificity obtained by EWS-DDA was the second highest among all of the five methods. For the case of N versus E.S in the genomics discovery set, EWS-DDA had the second best performance, which was slightly lower than DMNC. However, for the genomics validation set 2, EWS-DDA had the best performance of ROC, sum of specificity and sensitivity, S.E. and 95%CI for the datasets GSE62232 and GSE174570. For the case of HCC versus non-HCC and HCC versus CIR in the metabolomics discovery set and validation set, EWS-DDA also had the best values of ROC, sum of specificity and sensitivity, S.E. and 95%CI. The experimental results suggested that EWS-DDA has the power in potential biomarker discovery for HCC precise diagnosis.

## Discussion

Metabolic reprogramming plays an important role during the process of cancer initiation and progression and is used by cancer cells to meet their requirements for malignant proliferation and metastasis^21^. Change trends in cell metabolic activities can be viewed as cancer hallmarks for early diagnosis and treatment. Studies of fatty acid metabolism in clinical cancer management have attracted increasing attention. To our knowledge, although some previous studies have explored a single regulator of fatty acid metabolism in HCC, the integrated roles of genes and metabolites related to fatty acid metabolism are unknown. In this study, the network analysis algorithm EWS-DDA was applied to explore changes in genes and metabolites related to fatty acid metabolism in HCC initiation and progression, which can provide novel insights into fatty acid metabolism in HCC development and aid in an effective early diagnosis strategy in the clinic.

**Figure 6:**
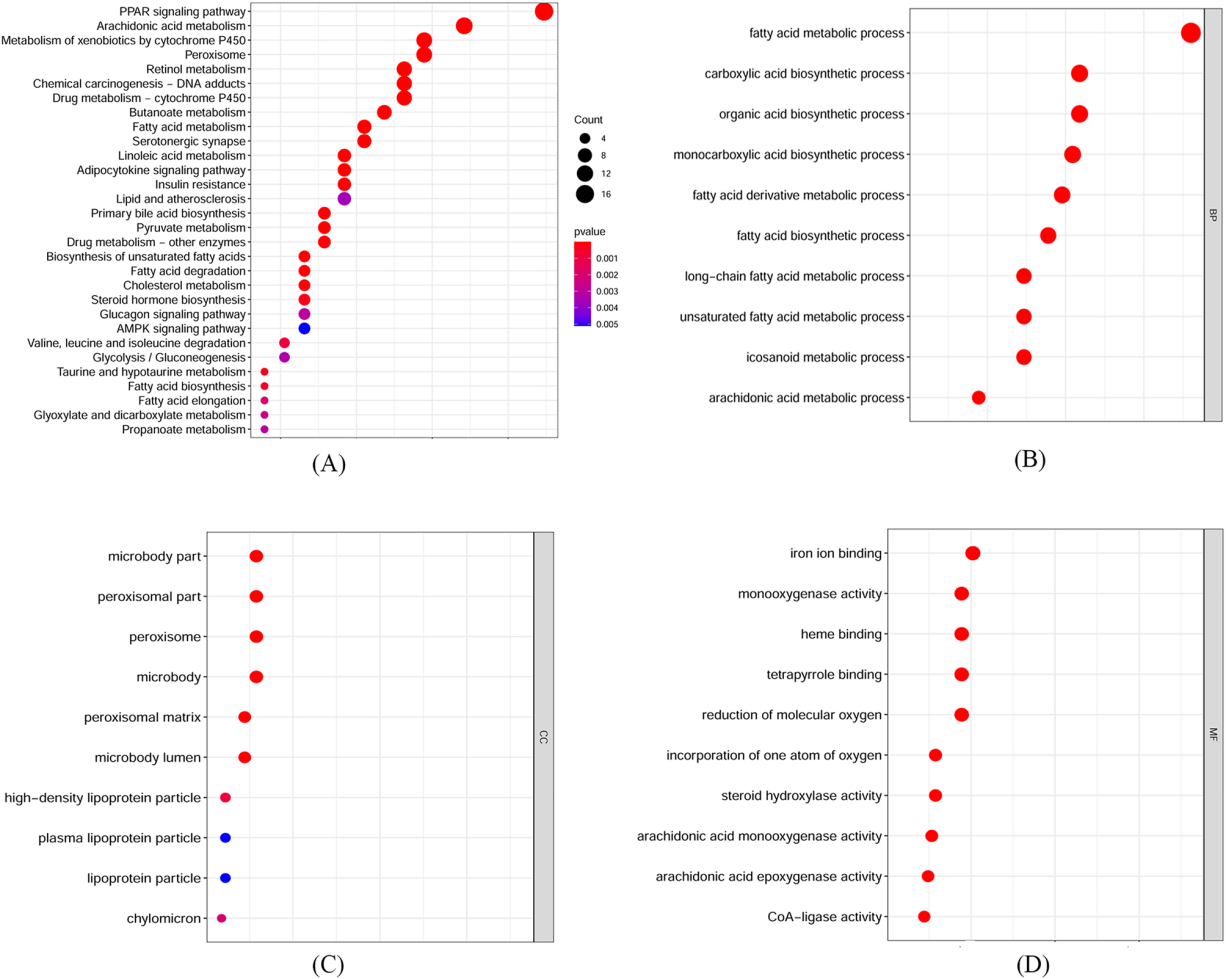
The results of enrichment analysis. (**A**) Bubble plots of KEGG. (**B**) Bubble plots of BP in GO. (**C**) Bubble plots of CCs in GO. (**D**) Bubble plots of MF in GO.

For the gene expression datasets, GO and KEGG enrichment analyses were used for functional annotation of these fatty acid metabolism-related genes. Figure 6 shows the bubble plots of KEGG and GO enrichment analyses of these genes. The KEGG results revealed the involvement of genes in many important pathways, including the PPAR signaling pathway, arachidonic acid metabolism, metabolism of xenobiotics by cytochrome P450, retinol metabolism and linoleic acid metabolism (see Fig. 6A). These biological activities are closely related to the known characteristics and pathogenesis of HCC. For example, PPAR regulates many important physiological activities, including invasion, immune tolerance, metabolism and inflammation, which were reported to be related to HCC progression^22,23^. Abnormal regulation of the PPAR signaling pathway generally occurs in the progression of tumorigenesis and cancer development^24,25^. Retinol metabolism is one of the crucial pathways related to the process of hepatic fibrosis, and enzymes of retinol metabolism are overexpressed in the early stage of HCC compared to precancerous lesions^26,27^. Therefore, altered retinol metabolism is a key signal for the progression of HCC. Linoleic acid, an important source of hydroperoxides, is one of the major fatty acids. Linoleic acid hydroperoxides can accelerate the cell cycle and reduce apoptotic activity by heme oxygenase 1 induction in hepatocarcinoma cells. Therefore, linoleic acid hydroperoxides can affect unaltered hepatic cells and the hepatocarcinogenesis process, which is a crucial driving force for carcinogenesis in the liver^28^. Cytochrome P450 plays an important role in the metabolism of endogenous and exogenous molecules. In-depth exploration of changes in cytochrome P450 enzyme activity is useful not only for facilitating the personalized treatment of HCC but also for defining key clinical information that contributes to HCC susceptibility^29^.

To better understand the potential function of these genes in HCC development, GO analysis was carried out. For the biological process (BP) results, fatty acid metabolic process, carboxylic acid biosynthetic process, organic acid biosynthetic process, fatty acid derivative metabolic process and fatty acid biosynthetic process were closely related to these genes (see Fig. 6B). The cellular components (CCs) of these genes were mainly involved in peroxisomes, microbodies, high-density lipoprotein particles, lipoprotein particles and chylomicrons (see Fig. 6C). In the molecular function (MF) analysis, these genes were mainly enriched in iron ion binding, monooxygenase activity, heme binding and arachidonic acid epoxygenase activity (see Fig. 6D).

The arachidonic acid metabolism pathway has been reported to be related to diverse human cancers^30^. The arachidonic acid metabolic enzyme PLA2G4F encodes a Group IV cytosolic phospholipase A2 with high specificity for arachidonic acid that is preferentially metabolized by cancer cells. Many studies have suggested that PLA2G4F is strongly implicated in the development and progression of cancer^30–32^. As one subtype of PPARs, PPARG is involved in the process of cellular differentiation and regulates lipid metabolism and energy balance, which can suppress HCC cell growth, angiogenesis, and migration^33,34^. PPARGC1A, a coactivator of PPARG, is an important regulator of energy metabolism. PPARGC1A is associated with HCC risk, which can influence susceptibility to HCC^35^. CYP2C8, a crucial antioncogene, plays an important role in oxidative metabolism and can metabolize many chemicals, such as steroids, arachidonic acids, retinoids and the anionic parts of some drugs. A previous study suggested that CYP2C8 influences HCC cell proliferation, clonality, migration and invasion via the PI3K/Akt/p27^kip1^ axis^36^. Therefore, CYP2C8 can be viewed as a potential biomarker for HCC diagnosis and prognosis. SCP2 can promote the uptake and metabolism of branched-chain fatty acids and direct cholesterol to cholesterol-rich cell membrane microstructures. SCP2 is closely related to the progression of cancer. Previous studies suggested that low expression of SCP2 can inhibit the proliferation of cancer cells by inducing autophagy^37^. ACOT is a superfamily of enzymes that plays an important role in lipid metabolism. ACOT controls one of the critical steps in catalyzing the hydrolysis of free fatty acids. Previous studies found that ACOT participates in the progression of tumorigenesis. Anchorage-dependent and anchorage-independent growth of HCC cell lines can be inhibited by ACOT knockdown. This growth inhibition was partially caused by free fatty acids, which suggests the importance of free fatty acids in cancer metabolism^38^. HMGCLL1, also known as HMG-CoA lyase-like 1, is a lyase activity enzyme located in the extramitochondrial region. HMGCLL1 polymorphisms have been reported to be relevant for cancer cell growth and viability^39^.

Three subtypes of fatty acids, namely, FFA, LPC and TAG, were selected as the crucial biological signals for HCC deterioration and early diagnosis. FFA has been previously reported to improve steatohepatitis and inhibit the development of HCC. Lipolysis contributes to cancer pathogenesis by altering FFA metabolism^38^. ACOT6 was identified in this study and is a lipolytic enzyme. ACOT knockdown inhibited the growth of HCC cell lines, which was rescued by the addition of FFA. This further suggests that FFA plays an important role in cancer metabolism. In this study, ACOT6 was selected from the HCC genomics dataset, and FFA was selected from the metabolomics dataset, which used different omics datasets to validate the theoretical analysis from previous studies. Therefore, the experimental results suggest that an in-depth evaluation of HCC development from different omics viewpoints helps to comprehensively understand the pathological alterations associated with HCC characteristics. LPC can be formed via the hydrolysis of phosphatidylcholine, which has an important role in cell signaling. Reduced circulating LPC levels have been reported to be associated with chronic liver diseases, and the levels of LPC species are also significantly altered in human HCC^40,41^. TAG is the major component of liver lipids. The changes in TAG levels were viewed as an index for evaluating metabolic syndromes by the WHO. Altered levels of TAG metabolism can play a role in the pathogenesis of HCC, which may be more effectively used for HCC risk assessment^42^. For the better and safer clinical application, further validation is still needed with a larger cohort of specimens.

In this study, the integration of the data from metabolomics and genomics was used for fatty acid metabolism studies to comprehensively understand the complex process of hepatocarcinogenesis. Dynamic changes in genes and metabolites related to fatty acid metabolism during HCC development were systematically explored by a dynamic network construction algorithm named EWS-DDA. Important potential gene and metabolite biomarkers were identified for early diagnosis and prognosis. The biological analysis suggested that the selected genes and metabolites were closely related to each other and coordinatively participated in the progression of tumorigenesis. Statistical analyses, including ROC, PCA, survival analysis and *t* test, demonstrated that the selected potential biomarkers had great power for clinical HCC applications, such as early diagnosis and prognosis. The experimental results demonstrated that the in-depth study of changes in genes and metabolites related to the fatty acid metabolism is a better way to comprehensively understand the complex biochemical processes associated with HCC development, contributing to improving the performance of clinical HCC management.

## Materials and methods

### HCC genomics datasets

The HCC genomics datasets consisted of a discovery set and a validation set, which were downloaded from The Cancer Genome Atlas (TCGA) and Gene Expression Omnibus (GEO) databases, respectively. The gene expression profiles in the discovery set contained normal samples and HCC samples (including stage I, stage II, stage III and stage IV). Based on functional analysis, 91 genes were found to be related to fatty acid metabolism. If a gene had missing values in a group, these values were replaced with 10% of the minimum nonzero value in that group. If the values of a gene were equal to zero in all samples in one group, the gene was excluded in this study. In total, 90 genes were used for subsequent network construction and analysis to reveal the role of fatty acid metabolism in HCC development. Three HCC gene microarray datasets (GSE116174, GSE62232 and GSE174570) constituted the validation set to further test the performance of potential biomarkers selected from the discovery set. GSE116174 contained only model samples with survival time and constituted validation set 1. This set was used to validate the prognostic values of the potential biomarkers. GSE62232 and GSE174570 contained the normal group and the model group and constituted validation set 2. This set was used to validate the diagnostic ability of the potential biomarkers. The detailed information of the HCC genomics datasets has been given in the supplemental information.

### HCC metabolomics datasets

The HCC metabolomics datasets analyzed by LC‒MS also contained a discovery set and a validation set^43^. Both normal and model groups were contained in the discovery set of HCC metabolomics. The collection of the time-series discovery set was conducted from week 8 to week 20 once every 2 weeks. Therefore, the model groups had 7 time points (*T*_1_–*T*_7_) representing the process of HCC progression. *T*_1_ (week 8) was the hepatitis stage, *T*_2_–*T*_4_ (weeks 10–14) was the cirrhosis (CIR) stage and *T*_5_–*T*_7_ (weeks 16–20) was the HCC stage. *T*_5_ was the key time point that signaled the onset of HCC. Similarly, there were also 7 time points in the normal group. Therefore, for the discovery set, there were 49 sera from 7 model rats in the model group and 70 sera from 10 control rats in the normal group. For the validation set, there were 36 sera from another 6 model rats that were sacrificed for histological examination with the affirmance of HCC at week 18. Therefore, 6 monitoring time points (*T*_1_–*T*_6_) were contained in the validation set. In this study, fatty acid metabolism-related metabolites, namely, DAG, FFA, LPC, LPE, PC, PE, PI and TAG, were studied in-depth to better understand the mechanistic changes in fatty acid metabolism during the process of HCC initiation and progression. The detailed information of the HCC metabolomics datasets has been given in the supplemental information.

### EWS-DDA

Molecular ratios can represent pathway reaction results that are essential for the implementation of various biological functions in systems biology. In tumor progression, the ratio relationships between molecules dynamically change, and there must be some change trends, namely, early warning signals, that play key roles in preventative measures. Therefore, EWS-DDA was proposed to construct a dynamic ratio network for the review of changes in assumed pathway reactions during tumor development, thereby aiding in the biochemical interpretability of pathogenic mechanisms^19^. In addition, the *t* statistic was considered in the construction of the network, which can define early warning signals by means of a data-driven method.

*F* = {*f*_1_,* f*_2_,…,* f*_*m*_} is the molecule set (i.e., genes or metabolites) and* X* = {*x*_1_, *x*_2_,…, *x*_*n*_} is the sample set, where *m* represents the number of features and *n* represents the number of samples. *C* = {*c*_1_, *c*_2_,…,* c*_*z*_} is the class label set that can represent the different stages of cancer development, in which *z* is the number of class labels. The molecule ratio *r*_*ij*_ can be represented as *f*_*i*_/*f*_*j*_. EWS-DDA used the shrunken centroid to reflect the differential change between the centroid of the molecule ratio *r*_*ij*_ for class *c*_*k*_ (i.e., stage *c*_*k*_) and its overall centroid. The shrunken centroid (*d*_*ijk*_) is calculated as follows:

1 $$d_{ijk} = \frac{{\mu_{ijk} - \mu_{ij} }}{{m_{k} (s_{ij} + s_{0} )}}$$

where *µ*_*ijk*_ is the average value of *r*_*ij*_ in the samples of class *k,* which is used to represent the centroid of *r*_*ij*_ for class *c*_*k*_, and *µ*_*ij*_ is the average value of *r*_*ij*_ in all the samples, which is used to represent the overall centroid of *r*_*ij*_. Therefore, the large value of *|d*_*ijk*_| can effectively reflect a relatively drastic biological change in the pathway reaction between *f*_*i*_ and *f*_*j*_ at stage *c*_*k*_ of cancer progression, contributing to the representation of cancer development to stage *c*_*k*_. In *d*_*ijk*_, *m*_*k*_**·s**_*ji*_ is equal to the estimated standard error of *µ*_*ijk*_-*µ*_*ij*_ when the parameter *m*_*k*_ is set as $\sqrt{{1}/\text{n} + {1}\text{/}{\text{n}}_{\text{k}}}$. *s*_*ij*_ represents the pooled within-class standard deviation for *r*_*ij*_. The parameter* s*_0_ can reduce the influence of gene ratios with different expression scale levels. Based on the suggestion from the reference,* s*_0_ is set as the median value of *s*_*ij*_ over the set of gene ratios. Therefore, comparing class *c*_*k*_ to the overall centroid, the shrunken centroid* d*_*ijk*_ is a *t* statistic for gene ratio *r*_*ij*_, which can be used for dynamic network construction^19,44^. In network *G*_*k*_ constructed by EWS-DDA, if *d*_*ijk*_ is larger than or equal to *ε* at the *k*th stage, *f*_*i*_ and *f*_*j*_ are linked by a red edge; if *d*_*ijk*_ is less than or equal to -*ε* at the *k*th stage, *f*_*i*_ and *f*_*j*_ are linked by a green edge.

### Network analysis

To identify warning signals, EWS-DDA focuses on topological changes in the crucial network signals of the early stage (without loss of generality, it is assumed to be class *c*_*q*_, 1 ≤ *q* ≤ *z*). Then, potential early diagnosis biomarkers can be identified based on the selected early warning network signals. The steps of network analysis are described as follows:

(I) Molecules in* G*_*q*_ have robust and definite relationships in stage *c*_*q*_; however, less definite relationships or even no definite relationships are presented in the other stages during the progression of cancer development.
(II) Based on the regular changes in network topology, network *G*_*q*_ can be viewed as an early network warning signal of cancer onset.
(III) To identify potential biomarkers for early clinical diagnosis of cancer, EWS-DDA ranks the nodes of *G*_*q*_ in descending order according to their degrees. Ratios between the top nodes and their first linking nodes are defined as crucial biological information for cancer clinical management.

### Network visualization and statistical analysis

Network visualization models were constructed by Cytoscape software (version 3.1.0) to directly show changes in molecular relationships in HCC progression. SIMCA-P 11.0 (Umetrics, Sweden) and SPSS 19 (IBM, USA) were applied for statistical analyses. The principal component analysis (PCA) model was constructed by SIMCA-P to represent the diagnostic ability of selected molecules. Receiver operating characteristic (ROC) analysis was implemented by SPSS, and index values, including the area under the ROC curve (AUC), specificity, sensitivity, standard error (S.E.) and confidence intervals (CIs), were applied to measure the effectiveness of the selected molecules. Multivariate Cox regression analysis can calculate the risk scores of recurrence for an individual by the points associated with each risk factor, and the median of risk scores was set as the threshold value to indicate high- and low-risk groups. Based on cumulative survival time, the Kaplan–Meier curve calculated by the results of multivariate Cox regression was applied to estimate the prognostic values implemented by R packages, in which the log-rank test was used for comparison between the low-risk and high-risk groups.

### The compared methods

Other state-of-the-art network construction and analysis methods, namely, DMNC, DNB-HC, ATSD-DN and MN-PCC were compared with EWS-DDA for the performance in early diagnosis biomarker discovery. Parameters for different methods in this study were set based on the suggestions from previous documents: the reference showed that when *te* was set as 100, DMNC can identify the network signals with the best discriminative ability for disease classification^45^; *num*, *α* and *ε* in DNB-HC were set as 1000, 0.05 and 0.8, respectively^46^; the parameter *ε* in ATSD-DN was set as 0.85 and the threshold value of PCC in MN-PCC was set as 0.7^43,47^.

### Functional analysis

To reveal potential HCC-associated biological functions that were regulated by the selected genes and metabolites, these molecules were mapped into known molecular sets with functional annotations. Gene Ontology (GO) and Kyoto Encyclopedia of Genes and Genomes (KEGG) analyses were used to better understand biological activities and for canonical pathway detection. Terms with the *p* value and *q* value < 0.05 in each biological process or pathway were considered significantly associated with HCC development and were presented in a bubble chart. R packages were used to implement these enrichment analyses.

### Supplementary Information

Supplementary Information.

## Data Availability

Genomics datasets can be downloaded from https://portal.gdc.cancer.gov and https://www.ncbi.nlm.nih.gov/geo. Metabolomics datasets are from “A New Strategy for Analyzing Time-Series Data Using Dynamic Networks: Identifying Prospective Biomarkers of Hepatocellular Carcinoma. *Sci. Rep.* **6**, 11, doi: 10.1038/srep32448 (2016)”.
