# Supplementary material for: Multiomics characterization of fatty acid metabolism for the clinical management of hepatocellular carcinoma
Source: Sci Rep. 2023 Dec 18;13:22472. doi: 10.1038/s41598-023-50156-7 (PMC10728109; doi:10.1038/s41598-023-50156-7)
Supplement: Supplementary file 1 — Supplementary Information. [file 41598_2023_50156_MOESM1_ESM.docx]

**Multiomics Characterization of Fatty Acid Metabolism** **for the Clinical Management of Hepatocellular Carcinoma**

Xin Huang^1,2,3*^ , Benzhe Su^4^, Mengjun Li^1^, Yang Zhou^5^, Xinyu He^6^

^1^ School of Artificial Intelligence, Anshan Normal University, Anshan, Liaoning, China.

^2^ Biomedical Engineering Postdoctoral Research Station, Dalian University of Technology, Dalian, Liaoning, China

^3^ Postdoctoral Workstation of Dalian Yongjia Electronic Technology Co., Ltd, Dalian, Liaoning, China

^4^ School of Computer Science and Technology, Dalian University of Technology, Dalian, Liaoning, China.

^5^ Ningbo Institute of Innovation for Combined Medicine and Engineering, Ningbo Medical Center Li Huili Hospital, Ningbo, Zhejiang, China

^6^ School of Computer and Information Technology, Liaoning Normal University, Dalian, Liaoning, China.

Corresponding Author:

Xin Huang

Pingan street, Anshan, Liaoning, 114007, China

Email address: huangxin0416@126.com


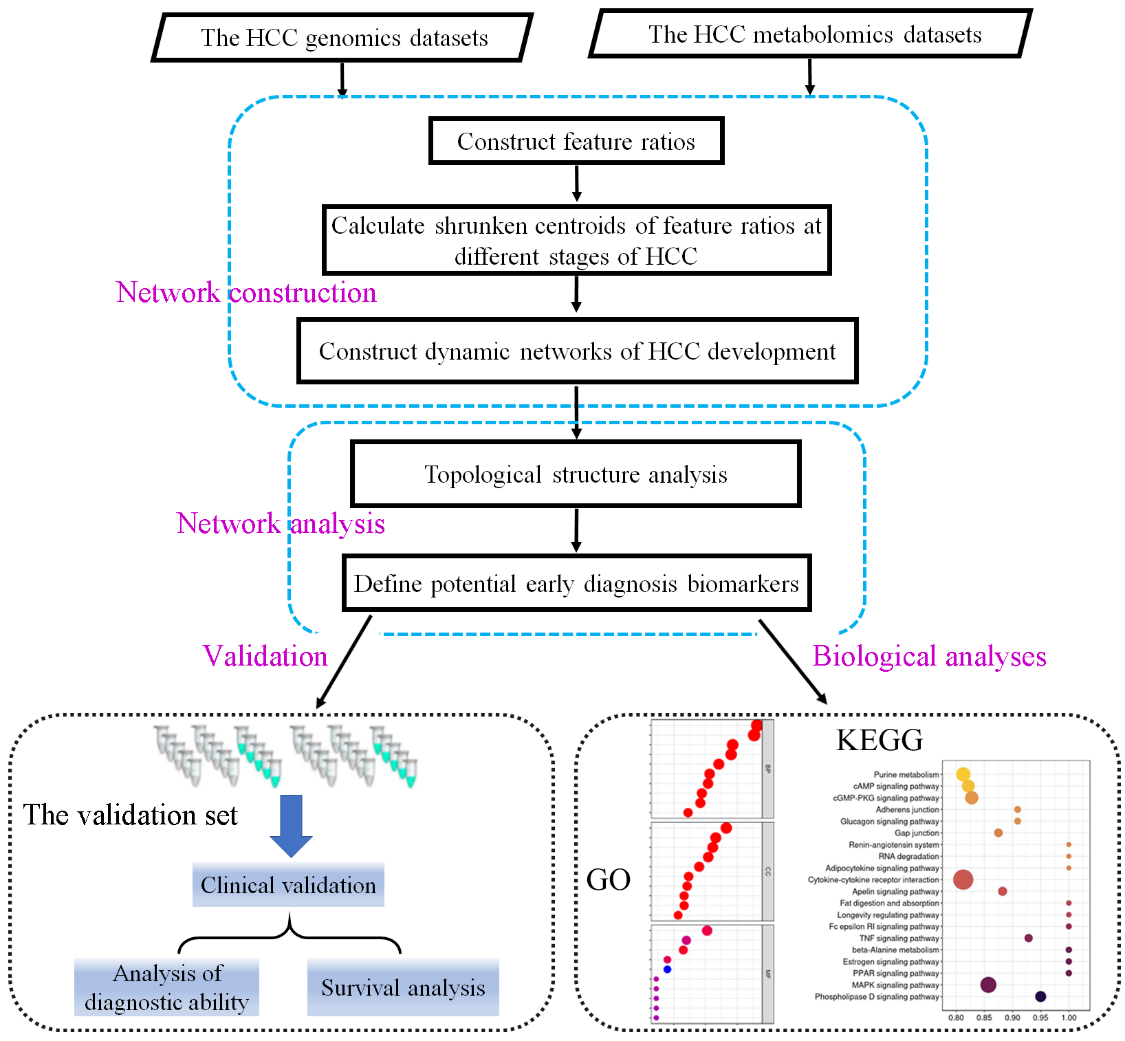


Figure S1 The comprehensive details on the procedures of this study.


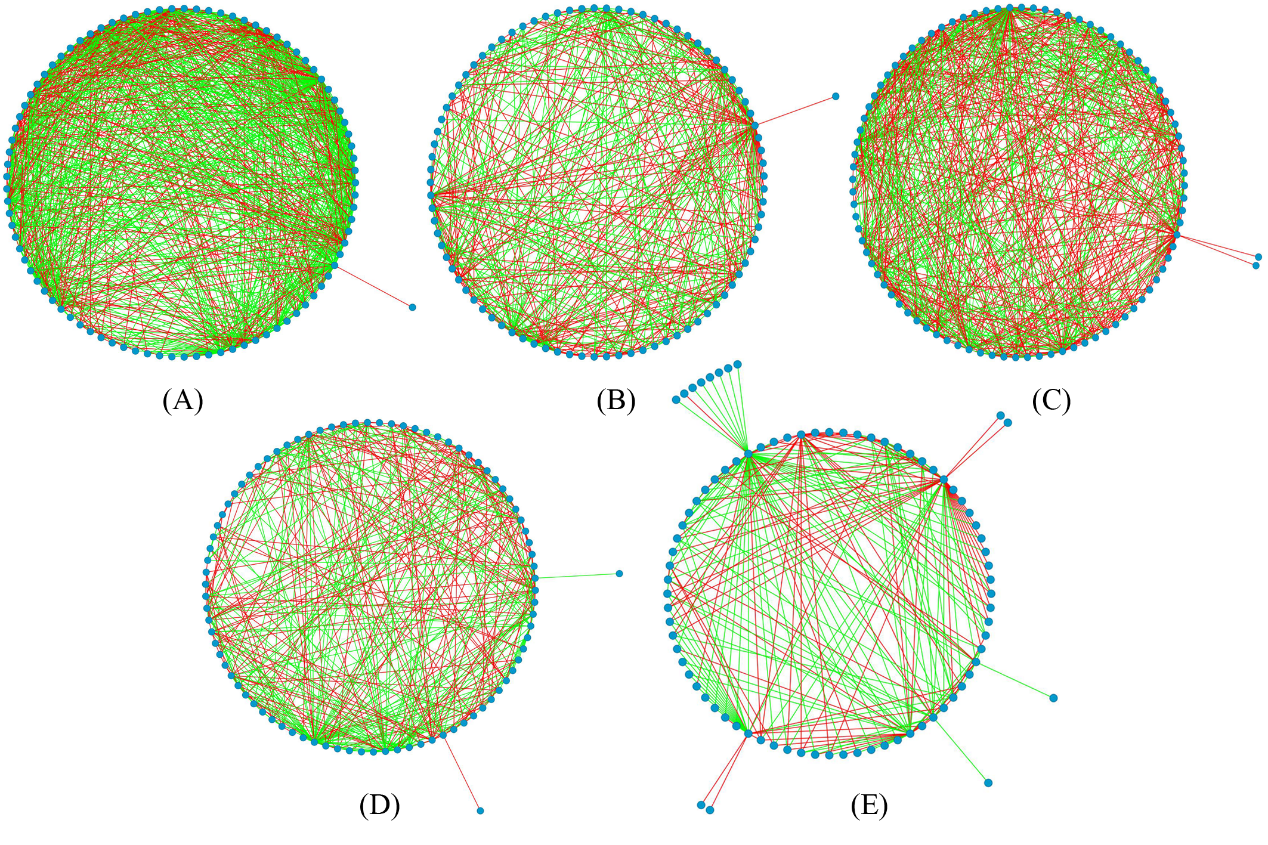


Figure S2 Dynamic gene networks constructed by EWS-DDA. (A)-(E) The topological structure of gene networks *G*_N_, *G*_I_, *G*_II_, *G*_III_, and *G*_IV_.


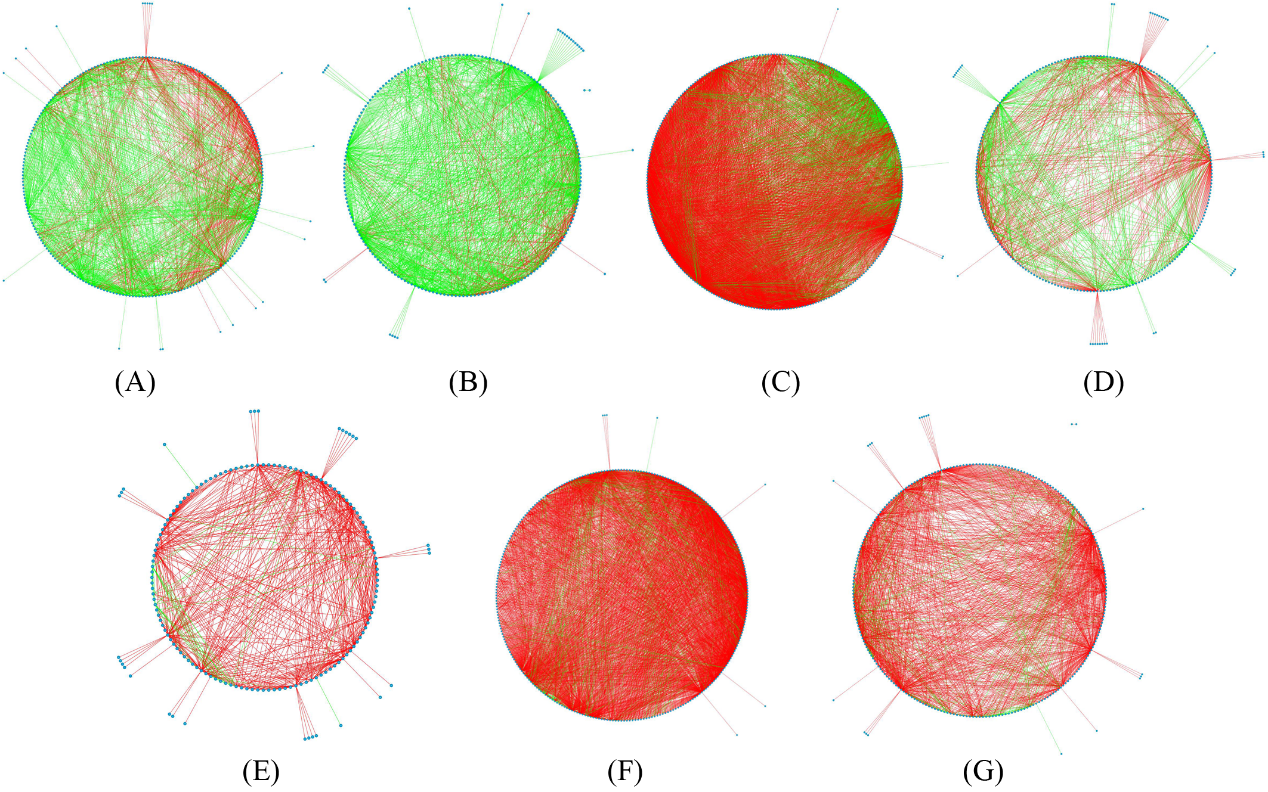


Figure S3 Dynamic metabolite networks constructed by EWS-DDA. (A)-(G) The topological structure of metabolite networks *DN*-*i* (1 ≤ *i* ≤ 7).

Table S1 Significant difference results of the selected six gene ratios

| Gene 1  (Numerator) | Gene 2 (Denominator) | N *vs*.  Stage I | N *vs*.  Stage II | N *vs*.  Stage III | N *vs*.  Stage IV | Stage I *vs*. Stage II | Stage I *vs*. Stage III | Stage I *vs*. Stage IV |
| --- | --- | --- | --- | --- | --- | --- | --- | --- |
| PLA2G4F | PPARGC1A | 3.51E-02 | 1.38E-02 | 2.56E-02 | 5.50E-04 | 1.08E-03 | 7.22E-03 | 1.26E-08 |
| CYP2C8 | SCP2 | 1.79E-06 | 2.95E-17 | 3.75E-17 | 1.11E-05 | 4.58E-02 | 8.91E-03 | 2.89E-01 |
| ACOT6 | HMGCLL1 | 1.93E-03 | 8.47E-04 | 3.53E-03 | 1.00E-03 | 2.19E-02 | 3.84E-01 | 4.52E-03 |
| CYP4A11 | HMGCLL1 | 5.31E-04 | 9.44E-04 | 4.46E-03 | 1.04E-03 | 2.10E-02 | 4.12E-02 | 8.56E-01 |
| INSIG1 | SCP2 | 1.42E-04 | 1.48E-03 | 2.96E-03 | 1.45E-05 | 4.32E-02 | 1.88E-03 | 7.62E-01 |
| PTGES | SCP2 | 1.50E-02 | 2.38E-02 | 4.25E-03 | 7.32E-10 | 8.75E-03 | 6.85E-03 | 3.86E-01 |

Table S2 Significant difference results of the selected ten metabolite ratios

| Metabolite 1  (Numerator) | Metabolite 1  (Denominator) | N16 *vs*. M16 | N18 *vs*. M18 | N20 *vs*. M20 | M14 *vs*. M16 | M14 *vs*. M18 | M14 *vs*. M20 |
| --- | --- | --- | --- | --- | --- | --- | --- |
| LPC 16:0 | FFA 22:5 | 1.29E-03 | 5.05E-06 | 1.83E-02 | 3.05E-02 | 2.78E-03 | 1.29E-02 |
| LPC 16:0 | FFA 20:3 | 1.53E-03 | 2.72E-05 | 3.88E-03 | 1.89E-02 | 9.37E-03 | 5.07E-03 |
| LPC 16:0 | TAG 58:10 | 2.81E-03 | 3.35E-05 | 1.93E-03 | 9.92E-03 | 4.20E-03 | 8.12E-03 |
| LPC 16:0 | FFA 19:0 | 3.83E-03 | 1.44E-03 | 2.77E-04 | 2.14E-03 | 1.72E-03 | 5.38E-03 |
| LPC 16:0 | FFA 17:0 | 4.46E-03 | 3.31E-04 | 8.43E-04 | 4.82E-03 | 2.92E-03 | 1.22E-02 |
| LPC 16:0 | FFA 17:1 | 5.06E-03 | 1.40E-03 | 5.46E-03 | 1.37E-02 | 1.16E-02 | 2.46E-02 |
| LPC 16:0 | FFA 20:2 | 5.56E-03 | 1.48E-04 | 8.73E-03 | 1.41E-02 | 1.32E-02 | 6.20E-03 |
| LPC 16:0 | FFA 15:0 | 1.08E-02 | 3.84E-03 | 2.14E-04 | 1.01E-02 | 1.38E-02 | 5.16E-03 |
| LPC 16:0 | PC 33:2 | 1.16E-02 | 2.28E-04 | 3.41E-04 | 2.43E-02 | 2.15E-02 | 2.81E-02 |
| LPC 16:0 | PI 36:3 | 2.72E-02 | 1.87E-03 | 1.24E-03 | 1.39E-02 | 9.98E-03 | 6.19E-03 |

**Detailed information of the HCC genomics and metabolomics datasets**

According to the clinical sample information from TCGA database, the genomics discovery set contained 50 normal samples, 171 stage I HCC samples, 86 stage II HCC samples, 85 stage III HCC samples and 5 stage IV HCC samples. For the genomics validation set 1, GSE116174 contained 64 HCC samples with survival time, which was used to validate the prognostic values of the selected potential biomarkers. For the genomics validation set 2 that was used to validate the diagnostic ability of the selected potential biomarkers, GSE62232 contained 10 normal samples and 81 HCC samples; GSE174570 contained 57 normal samples and 57 HCC samples.

For the metabolomics datasets, a total of 55 male Sprague-Dawley rats were enrolled in the present study at the age of 42 days (i.e., week 0). Then, after two weeks of adaptation, all rats were randomly divided into control (*n* = 10) and model (*n* = 45) groups, administrated with saline and diethylnitrosamine at 70 mg/kg body weight respectively via intraperitoneal injection. The injection was performed once a week between week 2 and week 11, and 14 rats from the model group died during the administration.

Histological examination was performed to monitor the progress of stepwise hepatocarcinogenesis based on the sacrifice of model rats, until all of the surviving animals (*n* = 10 for control group and *n* = 7 for model group) were finally sacrificed in week 20. Collected liver tissues were fixed in 10% buffered formalin and embedded in paraffin for histological examination, which confirmed that the diethylnitrosamine-induced hepatocarcinogenesis model was successfully produced in the present study.

The collection of time-series sera set was conducted from week 8 to week 20 once every 2 weeks (i.e., 7 monitoring time points). The discovery data included 10 rats from the control group and 7 rats from the model group. A total of 119 time-series sera were then collected from all 7 monitoring time points once every two weeks from week 8 to week 20. Thus, the number of the time points for the discovery set was 7. In the model group, the first time point *T*_1_ was week 8 and the 7th time point *T*_7_ was week 20. The serial progression of hepatocarcinogenesis was divided into three stages: week 8 (hepatitis stage, *T*_1_), weeks 10–14 (cirrhosis stage, *T*_2_–*T*_4_) and weeks 16–20 (HCC stage, *T*_5_–*T*_7_).

Furthermore, 36 sera from another 6 model rats were used for validation. These 6 rats were sacrificed for histological examination with the affirmance of HCC at week 18. Therefore, their sera were collected from 6 monitoring time points (i.e., *T*_1_–*T*_6_).
